# *Cripto* haploinsufficiency affects *in vivo* colon tumor development

**DOI:** 10.3892/ijo.2014.2412

**Published:** 2014-04-30

**Authors:** EMILIA GIORGIO, ANNAMARIA LIGUORO, LUCA D’ORSI, SARA MANCINELLI, ANTONIO BARBIERI, GIUSEPPE PALMA, CLAUDIO ARRA, GIOVANNA L. LIGUORI

**Affiliations:** 1 Institute of Genetics and Biophysics ‘Adriano Buzzati-Traverso’ (IGB), Consiglio Nazionale delle Ricerche (CNR);; 2 Istituto Nazionale per lo studio e la cura dei Tumori IRCCS ‘Fondazione G. Pascale’, 80131 Naples, Italy

**Keywords:** colon cancer, azoxymethane, *Cripto* heterozygous mice, glucose regulated protein-78

## Abstract

Colorectal cancer is one of the most common and aggressive cancers arising from alterations in various signaling pathways, such as the WNT, RAS-MAPK, PI3K and transforming growth factor-β (TGF-β) pathways. Cripto (also called Teratocarcinoma-derived growth factor), the original member of the vertebrate EGF-CFC family, plays a key role in all of these pathways and is deeply involved in early embryo development and cancer progression. The role of Cripto in colon and breast cancer, in particular, has been investigated, as it is still not clearly understood. In this article, we provide the first *in vivo* functional evidence of a role of Cripto in colon cancer development. We analyzed the effect of *Cripto* haploinsufficiency on colon tumor formation by treating *Cripto* heterozygous mice with the colonotropic carcinogen azoxymethane (AOM). Of note, in our model system, *Cripto* haploinsufficiency increased tumorigenesis. Moreover, we revealed a correlation between the differential AOM response found in wt and *Cripto^+/−^* mice and the expression levels of glucose regulated protein-78 *(Grp78)*, a heat shock protein required for Cripto signaling pathways. We hypothesize that the balance between *Cripto* and *Grp78* expression levels might be crucial in cancer development and may account for the increased tumorigenesis in *Cripto* heterozygous mice. In summary, our results highlight the heterogeneous effect of Cripto on tumorigenesis and the consequent high level of complexity in the Cripto regulatory pathway, whose imbalance causes tumors.

## Introduction

Colorectal cancer (CRC) is an important contributor to cancer mortality and morbidity, being one of the most prevalent and deadly cancers in the developed world, including Italy (1,2). Although a small subset of CRC cases are well-characterized hereditary syndromes, such as familial adenomatous polyposis (FAP) and hereditary non-polyposis colon cancer (HNPCC), the vast majority of CRCs are considered non-familial, occurring in individuals with heightened genetic susceptibility as a result of the interaction between multiple genes with low penetrance and environmental exposures (3). A long search has uncovered several genes and pathways which are important in the initiation and progression of CRC; these include the WNT, RAS-MAPK, PI3K and transforming growth factor-β (TGF-β) pathways (4,5). *Cripto* gene, involved in many decisions during early embryo development as well as in tumorigenesis, plays a key role in all of these pathways (6–9).

Cripto (or teratocarcinoma-derived growth factor) is the original member of the vertebrate EGF-CFC family of extracellular proteins, whose activity is fundamental during both embryonic and early postnatal life (10–12). *Cripto* is expressed very early during mouse embryogenesis, and it is involved in mesoderm formation, epithelial to mesenchymal transition (EMT) and the definition of the anterior-posterior axis (13,14). Cripto is a GPI-anchored protein (15) but can also act as a soluble factor (16). Cripto protein is an obligatory co-receptor for the TGF-β family members Nodal and growth differentiation factor (GDF) 1 and 3, enabling them to bind to Activin type receptorial complexes (8,17) and activate Smad-mediated gene expression (18). Apart from its co-receptor activity, Cripto is also able to antagonize the signaling of other members of the TGF-β family (i.e., Activins and TGF-β), due to a reduced ability of these ligands to form an active ActRII/ ActRI receptorial complex in the presence of Cripto (19,20). Cripto also acts via separate, non-overlapping mechanisms to enhance the canonical Wnt/β-catenin signaling pathway by binding to LRP5 and LRP6 co-receptors (21) and to activate ras/raf/MAPK and PI3K/Akt pathways via c-Src (9). More recently, novel Cripto-interacting proteins, including the chaperonin glucose regulated protein-78 (Grp78), have been identified (22). Grp78 forms a complex with Cripto at the cell surface, and this binding appears to be essential for all aspects of Cripto signaling (9,23).

High levels of *Cripto* mRNA and protein are expressed in a majority of human colon carcinoma cell lines and in 60–70% of human primary and metastatic colorectal tumors (24,25). *Cripto* expression has also been detected in several different types of human carcinomas, including breast, gastric, lung, pancreatic, bladder, cervical, skin and ovarian cancers (8,11), as well as in various colon, breast and nasopharyngeal tumor cell lines (26–29). In normal tissues, the expression of *Cripto* is absent or very low (30). Accordingly, low levels of Cripto protein were detected in the plasma of healthy volunteers, in contrast to patients with colon and breast carcinoma in whom a significant enhancement was found (30). *In vitro* functional studies on human cell lines have shown that Cripto causes the transformation of normal epithelial cells, promotes EMT and stimulates angiogenesis, cell proliferation and motility (31). Moreover, Cripto downregulation (at ∼50%) in human colon cancer cells drastically reduced their tumorigenicity (26). These data point to an oncogenic role for Cripto. Whereas the effects of Cripto overexpression on tumorigenesis has been studied *in vivo* in the breast of transgenic mice (32–35), as yet no data on the effect of reduced *Cripto* expression on tumor development *in vivo* has been reported.

In this study, we have analyzed for the first time how *Cripto* haploinsufficiency may affect *in vivo* cancer development by treating *Cripto* heterozygous mice (14,36) with the mutagenic agent azoxymethane (AOM) that exerts colonotropic carcinogenicity (37,38) and has been widely used to investigate the pathology and genetics of colorectal cancer in rodents (37,39). *Cripto^−/−^* mice die during early embryonic life (13,14) and therefore could not be utilized in this study. Our data provide the first *in vivo* functional evidence of a role of *Cripto* during colon cancer development and, in particular, of a positive effect of half *Cripto* gene dosage on tumorigenesis. These results reveal a dual effect of Cripto on tumor formation as well as a higher level of complexity in the Cripto regulatory pathway that affects tumorigenesis than has been previously shown. We suggest that the effect of Cripto on tumorigenesis strictly depends on the cellular context in which it acts and may be due to a different balance of the expression of *Cripto* and *Grp78*.

## Materials and methods

### Mice and carcinogen treatment

*Cripto* heterozygotes have been previously analyzed in both C57Bl6 mice and those with mixed genetic background (25% 129SvJ, 25% Black Swiss, and 50% C57B16); they were healthy and fertile and displayed no pathological conditions during their life span (14,36,40,41).

AOM was purchased from Sigma Aldrich, resuspended in PBS and stored at −80°C. To evaluate the response of *Cripto^+/−^* mice to chronic treatment with AOM, three-month-old female *Cripto^+/−^* mice (13) and wt littermates (7) were simultaneously treated intraperitoneally (i.p.) with AOM at a dose of 10 mg/kg body weight once a week for 6 weeks and sacrificed 30 weeks after the final AOM injection (37).

### TUNEL assay

To analyze the number of apoptotic cells in the colons of *Cripto^+/−^* and wt mice, two female C57Bl/6 wt and two *Cripto^+/−^* mice, three months of age, were administered a single i.p. dose of AOM at 10 mg/kg body weight, and sacrificed 6 h later. Two uninjected female wt mice of the same age were also analyzed. Colons were rinsed with ice-cold PBS, embedded in paraffin and sectioned. Serial sections were analyzed by the TUNEL method, using an *in situ* cell death detection kit, POD (Roche). Sections were deparaffinized, rehydrated and treated with 5 mg/ml proteinase K for 30 min at room temperature. Endogenous peroxidases were blocked by treatment with 0.3% H_2_O_2_. Digoxigenin conjugated nucleotides were placed directly on the sections in the presence of the terminal deoxynucleotidyltransferase enzyme in a humidified chamber at 37°C for 1 h. Sections were then incubated with converted-POD for 30 min at room temperature. After color development with 3,3′-diaminobenzidine and hydrogen peroxide, sections were observed by light microscopy (Leica DM6000 B). Images were captured, and apoptotic nuclei were counted in each crypt.

### Tumor characterization

After sacrifice, colons from cecum to rectum were removed, gently rinsed with ice-cold PBS to remove fecal material and then opened longitudinally. Pieces of tumor from 3 wt and 3 *Cripto^+/−^* mice were dissected and immediately frozen in liquid nitrogen for successive RNA extraction and analysis, whereas the remaining part of the samples was fixed in 10% formalin. All other colon samples were directly fixed in 10% formalin. The colon samples were dehydrated, embedded in paraffin and sectioned with a microtome to undergo various analyses. For microscopic examination, the paraffin-embedded sections were deparaffinized, rehydrated and stained with haematoxylin-eosin. The stained colon sections were carefully examined under the microscope to calculate tumor number and tumor area and to perform the stadiation of tumors. To calculate tumor area, the maximum colon section was chosen by looking at the sections under the microscope. The areas were summed for each mouse in order to calculate the total tumor area for a mouse; the total mean area of wt vs. *Cripto^+/−^* mice was then calculated. Sectional areas were calculated using the QWin-Leica program and were expressed as mm^2^. Histopathological analysis was performed independently by three pathologists who were blinded to the genotype.

### Immunohistochemical staining

For immunohistochemical staining, tissue sections were deparaffinized and rehydrated. Subsequently, the sections were heated in 10 mM sodium citrate pH 6.0 in the microwave twice, for 5 min each, to expose the antigens. Then, endogenous peroxidase activity was quenched with H_2_O_2_ 0.3% in methanol. Tissue sections were incubated at 4°C overnight with mouse monoclonal anti β-catenin antibody (Transduction Laboratories, Lexington, KY, USA) at 1:1000 dilution, or rabbit polyclonal anti Vascular Endothelial Growth Factor A (VEGF-A) antibody (Santa Cruz Biotechnology, Inc., Santa Cruz, CA, USA) at 1:100 dilution, or rabbit polyclonal anti GRP78 (Abcam) at 1:2000 dilution. The sections were then washed and incubated with biotinylated goat anti-mouse (DakoCytomation) 1:200 for anti-β-catenin and biotinylated goat anti-rabbit (DakoCytomation) 1:400 for anti-VEGF. After washing, the sections were incubated with avidin-biotin complex for 30 min using the Vectastain Elite ABC kit (Vector Laboratories Inc.). After color development with 3,3′-diaminobenzidine and hydrogen peroxide, sections were counterstained with hematoxylin. As a negative control, duplicate sections were immunostained without exposure to the primary antibody.

### Real-time RT-PCR analysis

RNA was extracted from normal and tumor tissues of both genotypes (wt and *Cripto^+/−^*) using TRIzol reagent (Invitrogen) and a glass-Teflon homogenizer. For each genotype, 3 different tumor samples and 2 normal colon samples were examined. All samples derived from different mice. Samples were incubated for 5 min at 15–30°C to permit the complete dissociation of nucleoprotein complexes, then, after the addition of 0.2 ml of chloroform, vigorously shaken for 15 sec, incubated at 15–30 °C for 2–3 min and centrifuged at 12,000 × g for 15 min at 4°C. RNA samples were precipitated using 0.5 ml of isopropyl alcohol, incubated at 15–30 °C for 10 min and centrifuged at 12,000 × g for 10 min at 4°C. RNA pellets were washed once with 75% ethanol and centrifuged at 7,500 × g for 5 min at 4°C. RNA was dissolved in RNase-free water and incubated for 10 min at 55°C. RNA was quantified by NanoDrop-1000 Spectrophotometer. cDNA synthesis was achieved by using the iScript^™^cDNA synthesis kit (BioRad). Real-time PCR was performed using three primer sets produced by QuantiTect Primer Assay (Qiagen) (QT00110075 for *Cripto*; QT00172361 for *Grp78*; QT00095242 for *Actin*). *Cripto* primers amplified a 104-bp fragment spanning exons 5 and 6; *Grp78* primers amplified a 140-bp fragment spanning exons 4, 5 and 6; *Actin* primers amplified a 149-bp fragment spanning exons 1 and 2. The reactions were conducted according to the iTaq^™^ Universal SYBR Green (BioRad) protocol. The PCR protocol involved a denaturation step (95° for 45 sec), followed by an amplification and quantitation program repeated 35 times (95° for 10 sec, 60° for 40 sec), and a melting curve program (60°C–95°C, with a heating rate of 0.5°C per second and continuous fluorescence measurement). The relative quantitation of gene expression was determined by the ΔΔ*C_t_* method. To normalize the output for each sample, the expression of *Cripto* and *Grp78* genes was divided by *Actin* gene expression. For each gene, the results are representative of two independent experiments.

### Statistical analysis

Results are presented as means ± SEM (structural equation modeling) of the mean for tumor multiplicity and tumor area and as means ± standard deviation of the mean for apoptosis and real-time analysis. The number of apoptotic cells, tumor multiplicity and tumor area among groups were compared by Student’s t-test. *Cripto* and *Grp78* expression levels among groups were analyzed with both Student’s t-test and Univariate analysis of variance (ANOVA). Tumor incidence was analyzed by Fischer’s exact probability test. Data were considered significant at p-value <0.05.

## Results

### Apoptosis detection after single AOM injection

**Figure 1.**
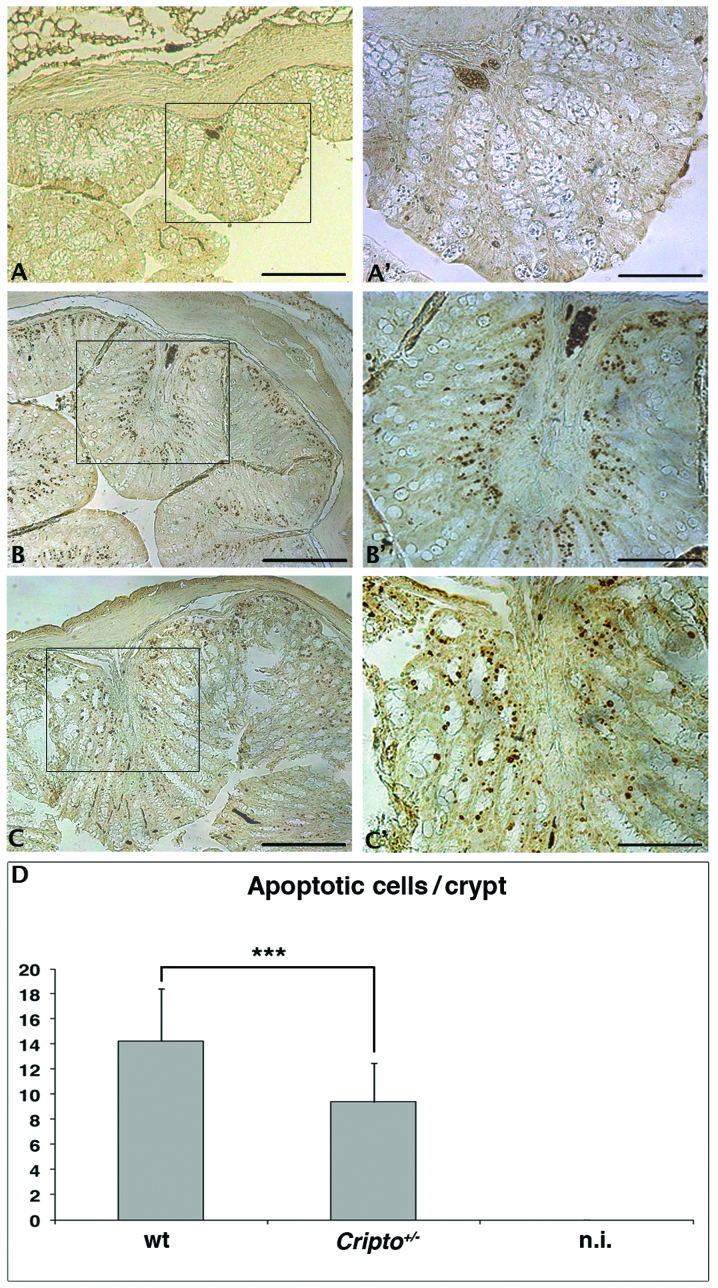
Comparative analysis of the apoptotic response in *Cripto* heterozygous and wt mice after single AOM injection. (A–C′) TUNEL analysis of colon cross sections from uninjected wt mice (A and A′) and both wt (B and ′) and heterozygous mice (C and C′), 6 h after AOM injection. Uninjected wt mouse colon does not show apoptotic cells (A and A′). The colon of wt mice (B and ′) shows more apoptotic cells than *Cripto* heterozygous mouse (C and C′). The average number of apoptotic cells inside a single crypt in the three groups of mice analyzed is reported in the graphic (D). The 3 sections and 9 crypts per section have been analyzed for each mouse, for a total amount of 27 crypts. (A′–C′) Magnifications of the areas enclosed in the boxes depicted in (A–C), respectively. Bar indicates 250 *μ*m (A–C), 125 *μ*m (A′–C′). (D) Data are shown as the mean ± SEM (bars). Comparisons were made to the corresponding controls (wt mice) using Student’s t-test, ^***^p<0.001.

First, we verified whether *Cripto^+/−^* and wt mice respond differentially to AOM. It has been shown that, following carcinogen treatment, the colonic epithelium undergoes cell growth arrest and apoptosis which facilitate the repair or elimination of genetically damaged cells (42). In particular, in the case of AOM, the maximum apoptotic death rate has been detected 6 h after single AOM injection (43). Therefore, we analyzed the number of apoptotic cells in the colon of C57Bl6 female *Cripto^+/−^* and wt mice by TUNEL, 6 h after single AOM injection (Fig. 1). Colons from untreated wt (Fig. 1A and A′) and *Cripto^+/−^* (data not shown) mice did not show apoptotic nuclei in colon crypts, whereas numerous apoptotic nuclei were detected in colons of both wt (Fig. 1B and ′) and *Cripto^+/−^* (Fig. 1C and C′) mice treated with AOM. We counted the number of apoptotic nuclei for the two genotypes, and we observed a reduced number of apoptotic cells in *Cripto^+/−^* mice compared to wt mice (Fig. 1D). These data suggest that *Cripto* haploinsufficiency is enough to alter the apoptotic response of colon cells to a short treatment with the AOM carcinogen. Surprisingly, *Cripto* heterozygosity causes a reduction of apoptotic cells.

### Analysis of colon carcinoma development following chronic AOM treatment

**Figure 2.**
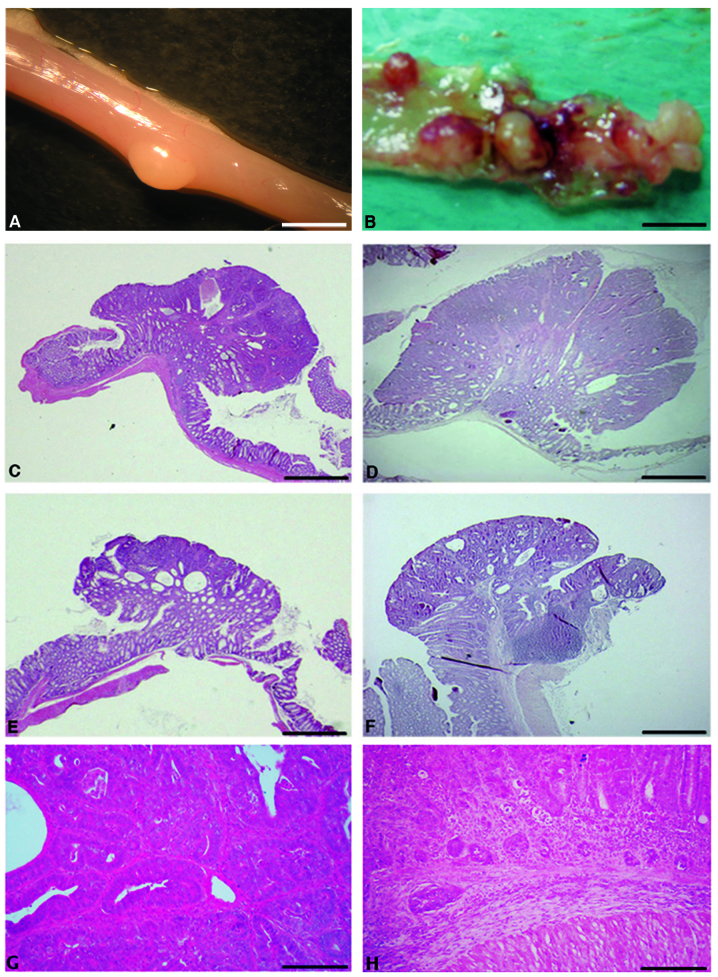
Macroscopic and microscopic analysis of colon tumors induced in wt and *Cripto^+/−^* mice. (A) A representative image of wt mouse colon opened longitudinally showing a single tumor. (B) A representative image of *Cripto^+/−^* mouse colon opened longitudinally showing several highly vascularized tumors of different size and with hemorrhagic areas. (C–F) Haematoxylin-eosin-stained cross sections of wt (C, E and G) and *Cripto^+/−^* (D, F and H) colon tumors. Bar indicates 4 mm (A), 5 mm (B), 0.5 mm (C-F) and 100 *μ*m (G and H).

**Figure 3.**
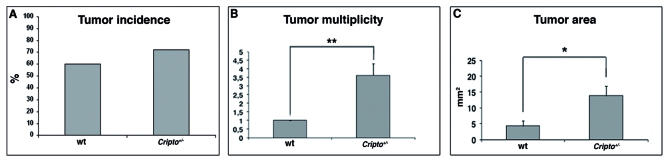
Comparison of tumor parameters in *Cripto^+/−^* and wt mice. *Cripto^+/−^* mice show comparable tumor incidence (A), but higher tumor multiplicity (B) and tumor area (C) than wt mice. (A–C) Data are shown as the mean ± SEM (bars). Comparisons were made to the corresponding controls (wt mice) using Fischer’s exact probability test for tumor incidence and Student’s t-test for tumor multiplicity and tumor area, ^*^p<0.05, ^**^p<0.01.

We evaluated the response of *Cripto^+/−^* mice to the chronic treatment with AOM described in Materials and methods. After sacrifice, we carefully dissected the mouse colons (Fig. 2A and B), and tumors were sampled, formalin-fixed and embedded in paraffin. Serial tumor sections were stained with haematoxylin-eosin (Fig. 2C–H), and tumor incidence (percentage of mice developing tumors), tumor multiplicity (number of tumors per mouse), tumor area per mouse and microscopic features were evaluated in both wt and *Cripto^+/−^* samples. Whereas tumor incidence did not vary significantly between *Cripto^+/−^* and wt mice (Fig. 3A), tumor multiplicity was significantly higher in *Cripto^+/−^* than in wt mice (1 vs. 3.6, p<0.01; Fig. 3B). Moreover, *Cripto^+/−^* mice showed higher values of tumor area than wt mice (4.3 mm^2^ vs. 13.8 mm^2^, p<0.05; Fig. 3C). Microscopic analysis revealed that all tumors in wt mice were adenomas with high grades of dysplasia (Fig. 2C, E and G), while in *Cripto^+/−^* mice we found adenocarcinoma (14%, Fig. 2H) in addition to adenomas with high grades of dysplasia (81%, Fig. 2D and F) and gastrointestinal intraepithelial neoplasia (GIN, 5%)

Altogether, these data demonstrate that *Cripto* heterozygous and wt mice respond differentially to long-term AOM treatment. In particular, *Cripto^+/−^* mice develop more numerous and larger colon tumors than wt mice, some of them being adenocarcinomas.

### Immunohistochemical characterization of colon tumors

**Figure 4.**
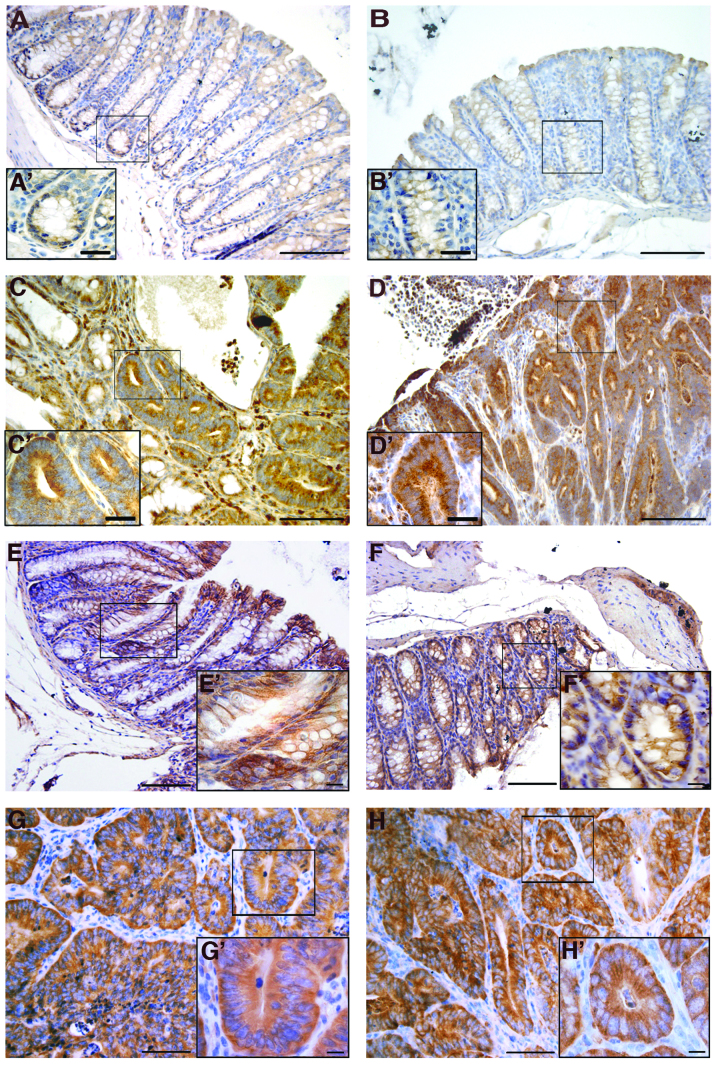
Immunohistochemical characterization of normal colons and colon tumors of wt and *Cripto^+/−^* mice. (A–D) VEGF immunolocalization. VEGF is weakly expressed in the normal colon epithelium of both wt (A and A′) and *Cripto^+/−^* (B and B′) mice, while it is strongly expressed in all colon tumors analyzed, without any significant difference between wt (C and C′) and *Cripto^+/−^* (D and D′) genotypes. (E–H) Immunohistochemical staining with β-catenin antibody. In the normal colon of both wt (E) and *Cripto^+/−^* (F) mice, β-catenin is localized at the cell-cell borders. (E′ and F′) Magnification of E and F, respectively, showing a single crypt with strong staining at the cell membranes. In colon carcinoma from wt (G) and *Cripto^+/−^* (H) mice, β-catenin is highly expressed and localized not only at the level of the plasmatic membrane, but also inside the cell. Both tumors are *in situ* carcinomas. All sections are transverse sections. (A′–H′) Magnifications of the areas enclosed in the boxes depicted in A–H. Bar indicates 100 *μ*m (A–H) and 5 *μ*m (A′–H′).

Several studies have implicated the VEGF in colon cancer angiogenesis (44). Cripto itself seems to have an important role in the multistep process of angiogenesis (31). For this reason, we analyzed VEGF expression in normal colons and colon tumors of both wt and *Cripto^+/−^* mice. Our results showed strong VEGF immunoreactivity in all colon tumors analyzed, independent of genotype (Fig. 4C, C′, D and D′), whereas VEGF was only weakly present in the normal colon epithelium of untreated mice (Fig. 4A, A′, B and ′) and near the tumors of injected mice (data not shown).

We also studied the immunolocalization of two other proteins related to the Cripto signaling pathway: β-catenin and Grp78. Cross-talk between Wnt/β-catenin and Cripto pathways has been widely demonstrated (45). β-catenin is also one of the most frequently mutated genes in AOM-induced colon carcinogenesis and plays important roles in the cadherin-mediated cell-cell adhesion system (46). The mutation causes the alteration of β-catenin cellular localization. β-catenin is normally located at the plasma membrane, but shifts to the cytoplasm and then to the nucleus during tumorigenesis (46). We used immunohistochemistry to examine the expression and distribution of β-catenin in normal colons from uninjected mice and in both normal colons and colon tumors of treated mice. In normal colon cells of both wt (Fig. 4E and E′) and *Cripto^+/−^* (Fig. 4F and F′) untreated mice, β-catenin was mainly localized at the cell-cell borders. In all of the adenocarcinomas analyzed, independent of genotype, stronger immunoreactivity for β-catenin was observed compared to the untreated mice, as well as a shifting of the signal to the cytoplasm (Fig. 4G, G′, H and H′). Finally, the localization of the β-catenin in the colonic epithelium close to tumors is also confined to the cell membranes (data not shown), as in the normal colons of the untreated mice. These data indicate that β-catenin localization changes between normal colons and colon tumors, but that the genotype of the mice does not significantly affect this localization pattern.

**Figure 5.**
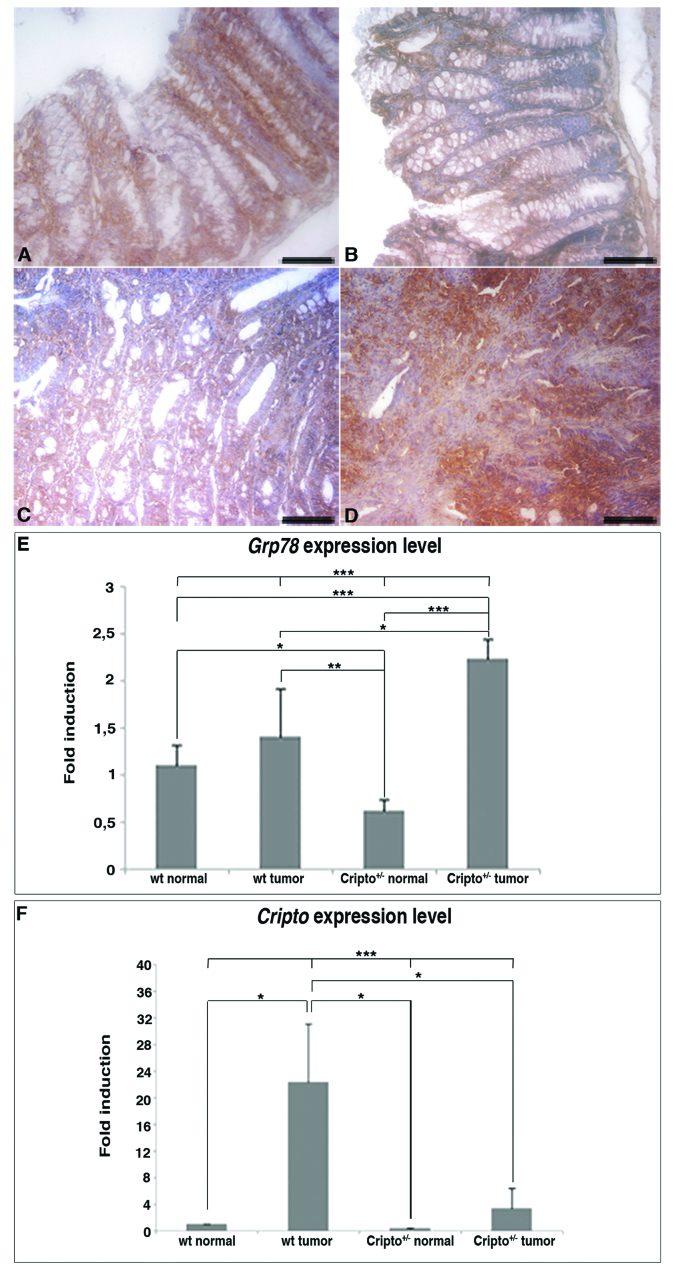
Grp78 expression analysis in normal colons and colon tumors of wt and *Cripto^+/−^* mice. (A–D) Grp78 immunohistochemical detection. Grp78 is expressed in normal colon epithelium (A and B) and colon tumor (C and D) of both wt and *Cripto^+/−^* mice. The strongest Grp78 staining is detected in Cripto+/− tumors. Bar indicates 100 *μ*m (A–D). (E and F) Comparison of Grp78 and *Cripto* expression levels in normal colons and in colon carcinoma of both wt and *Cripto^+/−^* mice. Histograms represent the real-time PCR values of *Grp78* (E) and *Cripto* (F) mRNA levels. Data are shown as the mean ± standard deviation. Significance of the results was evaluated using both Student’s t-test and ANOVA, ^***^p<0.001.

Last, we analyzed the expression of the heat shock protein Grp78 (Fig. 5), which is a fundamental player in all aspects of Cripto signaling via both TGF-β and Src/MAPK/PI3K pathways (9). Grp78 is also highly induced in a wide range of tumors and plays a critical role in tumor cell survival, tumor proliferation, angiogenesis and metastasis (47). Grp78 is expressed in normal colons (Fig. 5A and B) and colon tumors of both wt and Cripto heterozygous mice (Fig. 5C and D), but in *Cripto^+/−^* immunoreactivity in tumor samples is stronger than in normal colons (Fig. 5B and D).

### Expression analysis of Cripto and Grp78 genes

To confirm the Grp78 immunohistochemistry data, we evaluated the expression levels of *Grp78* by quantitative real-time RT-PCR. We also compared *Grp78* to *Cripto* mRNA levels in normal and tumor colon tissues of both genotypes (Fig. 5E and F). In agreement with immunodetection analysis, RT-PCR experiments showed that *Grp78* expression levels were comparable between tumors and normal tissues in wt mice, whereas in *Cripto* heterozygotes *Grp78* levels were higher in tumors than in normal colons. Moreover, the amount of *Grp78* expression was significantly higher in *Cripto^+/−^* than in wt tumors (Fig. 5E). On the contrary, the level of *Cripto* expression in wt mice was higher in colon tumors than in normal tissue as expected but, interestingly, in *Cripto^+/−^* mice, it did not vary significantly between tumors and normal colons (Fig. 5F). Moreover, Cripto expression level in *Cripto^+/−^* tumors was much lower compared to that of wt tumors (reduced to less than half).

In summary, our data show that AOM-induced colon tumorigenesis is more severe in *Cripto^+/−^* than in wt mice and is not accompanied by a significant increase in *Cripto* expression, while it is characterized by an increase in *Grp78* expression level.

## Discussion

*Cripto* expression has been described in a variety of tumors and cell lines (8,11). Downregulation experiments have been performed in some of these cell lines, such as colon and nasopharyngeal, showing a reduction of monolayer growth, soft agar cloning efficiency, matrigel invasion and cell proliferation (26,29), all suggesting an oncogenic role for Cripto. However, until now, there have been no reports on the effect of reduced *Cripto* expression on tumor development *in vivo*. We thus investigated how *Cripto* haploinsufficiency might affect tumor development, using as a model system Cripto heterozygous mice treated with AOM, which has a specific colonotropic effect. Surprisingly, we found that *Cripto* heterozygotes have a higher susceptibility to AOM than wt mice with respect to the development of colon cancer. *Cripto* haploinsufficiency increases mouse tumor size and multiplicity, though it does not significantly affect tumor incidence. The increased tumor size and multiplicity found in *Cripto* heterozygous mice correlate well with the reduction of the apoptotic response to short AOM treatment. Our results show, for the first time, that a reduction in *Cripto* expression levels may be associated with an increase in tumor parameters.

The other *in vivo* studies published to date on the role of Cripto in tumorigenesis regard two mouse models in which *Cripto* was overexpressed in the mammary gland (32–35). Both studies have shown that mammary-specific over-expression of *Cripto* causes the development of mammary tumors in a percentage of multiparous aged female FVB/N mice (33% for Wechselberger and coauthors; 55% for Sun and coauthors). However, the latency period (12–20 months) was very long compared, for example, to Wnt-1 transgenic mice, which develop mammary tumors with a median latency of 6 months (48). This suggests that the overexpression of *Cripto* by itself is not sufficient to induce tumorigenesis, but that additional genetic alterations are required. It is noteworthy that Sun and coworkers have reported that 66.7% of multiparous heterozygous transgenic females vs. 45% of multiparous homozygous transgenic females develop mammary tumors, suggesting that the relation between *Cripto* expression levels and tumor development is not so obvious. Moreover, *Cripto* overexpression is also able to increase the apoptotic rate during mammary gland involution (34). To complicate the scenario, a loss of heterozygosity (LOH) at Chromosome 3p21.3, where Cripto is localized (49), has also been shown in a wide spectrum of human cancers, including lung (50), breast (51), nasopharyngeal (52) and kidney (53).

Cripto modulates the signaling of several TGF-β ligands, such as Nodal, GDF-1 and GDF-3, for which variable and even opposing effects on cellular proliferation and apoptosis have also been described (9,54). It has been shown that the different effects of TGF-β ligands on cell proliferation depend on the cell type and the cellular context (9,54). The cellular context also seems to be fundamental for Cripto function. We analyzed the expression of three molecules, VEGF, β-catenin and glucose regulated protein-78 (Grp78), which interact with the Cripto pathway and are also deeply involved in colon tumorigenesis. No significant differences between wt and *Cripto* heterozygotes have been detected by immunohistochemistry with both anti VEGF and anti-β-catenin antibodies. In contrast, Grp78 expression varies differentially between normal colons and colon tumors, depending on the genotype. By means of real-time RT-PCR, we compared the expression levels of *Cripto* and *Grp78* genes in normal and colon tumor tissues of both wt and *Cripto^+/−^* mice. In wt mice, we found, as expected, a significant increase in *Cripto* expression level but not in Grp78 expression level in tumor samples compared to normal colons. On the contrary, in *Cripto^+/−^* mice, we detected no significant variation in *Cripto* expression but a higher *Grp78* expression in colon tumors than in normal tissue.

Grp78 forms a complex with Cripto at the cell surface, and this interaction appears to be essential for all aspects of Cripto signaling via both TGF-β and Src/MAPK/PI3K pathways (9). *Grp78* expression has been widely associated in the literature with tumorigenesis (9). Notably, *Grp78* heterozygosity affects transgene-induced mammary tumor development, prolonging the latency period and inhibiting tumor growth, even though it does not affect tumor incidence (55). Therefore, an increase in *Grp78* expression could account for the phenotype detected in the *Cripto^+/−^* mice following AOM treatment that, similarly, is characterized by the same tumor incidence as wt mice, but with increased tumor multiplicity and size. As Grp78 is a chaperone, involved in many different signaling pathways, a deregulation of its expression might have a stronger effect on tumor phenotype than a reduction in *Cripto* expression. In other words, the tumorigenic effect due to the increase in *Grp78* expression *in vivo* would be stronger than the opposite effect due to *Cripto* haploinsufficiency. Furthermore, the inability of *Cripto* heterozygotes (due to the loss of one *Cripto* allele) to reach a threshold level of *Cripto* expression following activation of an AOM-induced tumorigenic pathway may cause the upregulation of *Grp78* expression level through a negative feedback loop. Due to the opposite effect of *Cripto* down-regulation in colon cancer cell lines (26), *in vitro* experiments might not easily help in dissecting the underlying mechanism regulating *Cripto* and *Grp78* expression.

In summary, we show for the first time that *Cripto* haploinsufficiency may be associated with increased tumorigenesis, suggesting that the effect of Cripto on tumor development is more complex than previously shown and may strongly depend on the cellular context. Moreover, we propose that the balance between Grp78 and Cripto expression is a promising regulative factor in tumor development. It would be interesting to investigate the expression levels of *Grp78* in other tumor model systems in which Cripto expression is dysregulated, to determine whether this scenario is specific to colon cancer or, more probably, can be generalized to the other types of tumors.
